# Gestational age as predictor of postoperative prognosis in neonates with pulmonary atresia with intact ventricular septum undergoing biventricular repair

**DOI:** 10.3389/fcvm.2026.1813144

**Published:** 2026-06-22

**Authors:** Shuo Chen, Ziqin Zhou, Luoming Hu, Jiazichao Tu, Weimin Chen, Miao Tian, Xiaohua Deng, Xiaohua Li, Zhanhao Su, Jimei Chen

**Affiliations:** 1 Department of Cardiac Surgery, Guangdong Provincial People’s Hospital, Southern Medical University, Guangzhou, China; 2 Guangdong Cardiovascular Institute, Guangdong Provincial People's Hospital, Guangdong Academy of Medical Sciences, Guangzhou, China

**Keywords:** complex congenital heart disease, gestational age, prognosis, pulmonary atresia with intact ventricular septum, threshold effect

## Abstract

**Background:**

Pulmonary atresia with intact ventricular septum (PA/IVS) is a rare critical congenital heart defect. Although biventricular repair is reserved for those with adequate right ventricular development, significant outcome variability persists even among anatomically similar patients, implicating non-structural determinants of surgical recovery. Gestational age has been recognized as a prognostic factor in various congenital heart diseases, yet its specific impact on short-term outcomes after biventricular repair for PA/IVS remains inadequately characterized.

**Methods:**

This retrospective cohort study enrolled 97 consecutive pediatric patients with PA/IVS who underwent biventricular repair at a single tertiary referral center from 1999 to 2024. Participants were categorized as preterm or term according to standard gestational age criteria.

**Results:**

The cohort comprised 21 preterm infants and 76 term infants. Multivariable analysis showed each additional gestational week reduced adverse outcome risk by 45% (OR 0.55, 95% CI 0.41–0.75). Term infants demonstrated significantly lower risk vs. preterm infants (OR 0.14, 95% CI 0.04–0.46). Restricted cubic spline regression revealed a nonlinear threshold effect (*P*-nonlinear=0.047) with an inflection point at 37.6 weeks (95% CI 36.6–38.7). Beyond this threshold infants had a 92% lower risk compared with those under 37.6 weeks (OR 0.08, 95% CI 0.02–0.42).

**Conclusions:**

This study establishes a gestational age threshold effect for short-term outcomes in PA/IVS patients undergoing biventricular repair. Extending gestation beyond 37.6 weeks significantly improves short-term outcomes in PA/IVS patients undergoing biventricular repair, providing crucial evidence-based guidance for perinatal management.

## 1 Introduction

Pulmonary Atresia with Intact Ventricular Septum (PA/IVS) is a rare and critical congenital heart defect, with an estimated incidence of 7.1–8.1 per 100,000 live births (1). Although staged surgical palliation has significantly improved survival (2), perioperative complications remain a significant clinical challenge. Approximately 10%–30% of infants experience adverse events, often resulting in prolonged intensive care, extended hospitalization, or death (3, 4).

Current management is primarily dictated by anatomical assessment of right ventricular (RV) morphology, which determines candidacy for biventricular (BiV), one-and-a-half, or single-ventricle pathways (5). For patients with adequate RV size, BiV repair is the intended strategy; those with severe RV hypoplasia are directed toward single-ventricle palliation. However, even among patients selected for BiV repair with comparable RV anatomy, postoperative outcomes vary substantially—suggesting that non-anatomical factors also influence surgical recovery in this pathway.

Emerging evidence identifies gestational age (GA) as an independent prognostic factor in congenital heart disease outcomes (6, 7). Steurer MA et al. incorporated GA and birth weight into outcome predictions for neonatal heart surgery in neonate with CHD (8). Some study further identified GA as an independent predictor of five-year mortality following pediatric cardiac surgery (9). Despite these findings, the impact of GA on outcomes specifically in PA/IVS remains incompletely characterized. Existing risk stratification models fail to adequately incorporate GA, which may limit their clinical applicability in guiding preoperative decision-making for this high-risk population. This study aims to facilitate the integration of GA into preoperative risk assessment, thereby supporting more individualized intervention timing and optimized perioperative management in infants with PA/IVS undergoing biventricular repair.

## 2 Methods

### 2.1 Ethics statement

The study was approved by the local ethics committee (KY2024-745-02) and conducted in accordance with the Declaration of Helsinki, with written informed consent obtained from all participants.

### 2.2 Study population

This retrospective cohort study included 97 consecutive pediatric patients with PA/IVS who underwent bi-ventricular repair at Guangdong Provincial People's Hospital between June 1999 and June 2024. The diagnosis of PA/IVS was established in accordance with the echocardiographic guidelines for congenital heart disease published by the American Society of Echocardiography (ASE) (10). To ensure a homogeneous cohort and minimize confounding from distinct pathophysiological entities, we restricted inclusion to patients with isolated PA/IVS. PA/IVS patients with concurrent complex intracardiac abnormalities, like Transposition of great artery and Ebstein anomaly, were excluded from this study. Additionally, to guarantee the integrity and completeness of the multivariable analyses, only patients with comprehensive baseline clinical data were included. Patients were excluded if >20% of baseline data were incomplete or if they experienced immediate loss to follow-up after diagnosis. Data were retrieved from the institutional follow-up database.

### 2.3 Research variable

This retrospective cohort study primarily examined the association between GA and postoperative adverse outcomes. Patients were categorized into preterm (*n* = 21, GA < 37 weeks) and term (*n* = 76, GA ≥ 37 weeks) groups based on GA. Short-term adverse outcomes were defined as the occurrence of prolonged hospital stay (>30 days), prolonged ICU stay (>15 days), or any major post-surgery adverse event (Table 1). For consistency, all events are assessed during the hospitalization or within 30 days postoperatively. This composite endpoint was constructed based on clinically relevant complications and has been consistently employed in prior studies of neonatal cardiac surgery (11). Long-term outcomes were assessed using a composite endpoint comprising mortality, conversion of repair strategy, or surgical reintervention. Outcome definitions were aligned with established consensus guidelines to ensure validity and comparability (4, 12).

**Table 1:** Definition of short-term adverse prognostic events.

**Adverse Prognostic Event**	**Definition**
Prolonged Hospital Stay	Postoperative hospital stay > 30 days
Prolonged ICU Stay	Postoperative ICU stay > 15 days
Definition of Major Adverse Events	
Death	All deaths occurring during hospitalization or within 30 days after surgery
Malignant Arrhythmia	Hemodynamically unstable arrhythmia (e.g., complete heart block, ventricular fibrillation)
Postoperative Reintervention	Unplanned surgical procedure during same hospitalization
Diaphragmatic Paralysis	Impaired diaphragmatic motion due to phrenic nerve injury, confirmed radiographically.
Multiple Organ Dysfunction Syndrome	Concurrent dysfunction of ≥2 organ systems requiring supportive treatment

Demographic and clinical characteristics included birth weight, preoperative oxygen saturation, and presence of associated cardiac anomalies. Birth weight, obtained from obstetric records or parental report and classified as low (≤2,500 g) or normal (>2,500 g). Right ventricular morphological parameters were echocardiographically measured prior to surgery, including RV dimensions, tricuspid valve Z-score, and degree of RV hypoplasia graded by the modified Bull's classification. PGE1 exposure was categorized by cumulative dosage intensity. The detailed notes of important variables is in Supplement materials. Surgical variables were obtained from original operative records.

### 2.4 Operation strategies and techniques

Indications for a B-T shunt include: (1) severe cyanosis (oxygen saturation <70%) in the neonatal period with duct-dependent pulmonary circulation; (2) significantly hypoplastic right ventricle unsuitable for initial BiV repair; (3) tricuspid valve annulus z-score <–2.5 with severe tricuspid regurgitation; (4) clinically unstable neonates requiring palliative stabilization. After shunt placement, patients are followed; conversion to definitive repair is considered when body weight reaches 8–12 kg, right ventricular diameter increases ≥20%, tricuspid z-score improves by ≥1 SD, and clinical status is stable (usually 6–24 months post-shunt). Four surgical approaches are used for biventricular circulation.

Modified Blalock-Taussig shunt: Under heparinization, the right subclavian artery and pulmonary artery were clamped. An end-to-side anastomosis was performed using a graft with continuous suture. After clamp release, anastomotic sites were inspected and confirmed secure.

Transcatheter pulmonary valve dilation: Via femoral venous access, a catheter was advanced to the atretic pulmonary valve. A radiofrequency wire perforated the valve under fluoroscopy, then exchanged for a guidewire. A balloon was advanced and inflated across the valve until the waist resolved. Post-dilation angiography confirmed adequate opening before sheath removal.

RVOT reconstruction: Through median sternotomy with cardiopulmonary bypass, the main pulmonary artery was incised and valve leaflets divided. A right ventriculotomy was made and obstructive muscle bundles excised. The RVOT and pulmonary artery were augmented with a pericardial patch using continuous suture. Tricuspid valve competence was assessed by saline testing before weaning from bypass.

Open pulmonary valvotomy: Via median sternotomy, the distal pulmonary artery was controlled and a longitudinal arteriotomy performed. Adherent valve leaflets were incised and dilated to approximately 3.5 mm, then the arteriotomy closed with continuous suture.

Hybrid off-pump procedure: Through median sternotomy, purse-string sutures were placed on the right ventricular free wall. Under transesophageal echocardiography, the atretic valve was perforated and balloon-dilated to establish antegrade flow. The access site was secured with pre-placed sutures and the wound closed.

### 2.5 Statistical analysis

Continuous variables were expressed as mean ± SD or median (IQR) and compared using *t*-test or Mann–Whitney *U*-test; categorical variables as *n* (%) using *χ*^2^ or Fisher's exact test. Logistic regression assessed the association between GA/birth weight and short-term adverse outcomes. We applied the EPV principle (≥10 events/variable) to avoid overfitting, adjusting solely for right ventricular hypoplasia and prenatal diagnosis in Model 2 and for GA and right ventricular hypoplasia in Model 4 (Model 1 and Model 3: unadjusted). Model discrimination was evaluated by AUC. Nonlinearity was explored using restricted cubic splines without additional covariates owing to sample size constraints. If the likelihood ratio test suggested nonlinearity (*P* < 0.05), a two-piecewise logistic model was fitted to identify thresholds. Subgroup analyses used Model 2, with interactions tested and displayed as forest plots. For long-term composite outcomes, Cox models with the same adjustments were applied; proportional hazards assumption checked via Schoenfeld residuals. Missing data were handled by multiple imputation (mice, R; 5 imputed datasets). Pooled estimates from the imputed data (original *N* = 97) are reported. Analyses used SPSS 27.0.1 and R 4.0.2; two-sided *P* < 0.05 was significant.

## 3 Results

### 3.1 Baseline clinical characteristics

The cohort comprised 97 neonates with PA/IVS, categorized into preterm (*n* = 21) and term (*n* = 76) groups (Table 2, Supplementary Table S1). Preterm infants had a mean GA of 34.14 ± 2.03 weeks, compared to 39.08 ± 1.17 weeks in the term group (*p* < 0.001). The preterm and term groups were well-balanced in terms of key baseline morphological and clinical characteristics (all *p* > 0.05). Preterm groups had a mean first treatment age of 66 ± 59 days, compared to 72 ± 176 days in term groups (*p* = 0.803). We additionally compared the first treatment age between the low birth weight group (*n* = 19) and the normal birth weight group (*n* = 78). Low birth weight infants (<2,500 g) had earlier first treatment age (39 ±  56 days) than normal birth weight infants (79 ± 173 days), but this difference also did not reach statistical significance (*p* = 0.095). Over the long term, no significant differences were observed between the preterm and full-term groups in mortality, final circulation type, reintervention rates, or adverse outcomes (all *p* > 0.05). As expected, preterm infants had higher prevalence of low birth weight (*p* < 0.001), elevated prenatal diagnosis rate (*p* < 0.001) and smaller body surface area (*p* = 0.028). The tricuspid annulus was also smaller in the preterm group (*p* = 0.002). The composite endpoint occurred in 27.8% of patients (27/97), with a significantly higher incidence in preterm vs. term infants [57.1% (12/21) vs. 19.7% (15/76); *P* = 0.037]. Among the events, death (29.6%), postoperative reintervention (18.5%), and MODS (14.8%) were most common (Table 3).

**Table 2:** Patient demographics and baseline characteristics.

Characteristic	Gestational Age Group		*p*-value
	˂37 weeks	≥37 weeks	
	*N* = 21	*N* = 76	
Perioperative Characteristic			
Admission year, *n* (%)			0.465
2000–2010	0 (0.0%)	7 (9.2%)	
2010–2020	17 (81.0%)	56 (73.7%)	
2020–2024	4 (19.0%)	13 (17.1%)	
Gender, *n* (%)			0.909
Female	8 (38.1%)	30 (39.5%)	
Male	13 (61.9%)	46 (60.5%)	
Low birth weight, *n* (%)	10 (47.6%)	9 (11.8%)	<0.001
Body surface area, m^2^	0.20 ± 0.05	0.23 ± 0.06	0.028
Prenatal diagnosis, *n* (%)	11 (52.4%)	14 (18.4%)	<0.001
Gestational age, weeks	34.14 ± 2.03	39.08 ± 1.17	<0.001
First treatment age, days	66 ± 59	72 ± 176	0.803
Echocardiographic Measurements			
TV Annulus, mm	9.8 ± 2.8	12.3 ± 3.5	0.002
TV z-score	−1.20 ± 1.71	−0.64 ± 1.62	0.187
TV/MV ratio	0.77 ± 0.23	0.86 ± 0.22	0.102
RV width, mm	14.0 ± 4.1	13.6 ± 4.5	0.703
PA annulus, mm	8.00 ± 1.98	8.14 ± 2.29	0.776
RA dimension, mm	22 ± 4	24 ± 8	0.043
RVOT, mm	7.7 ± 3.0	8.0 ± 3.3	0.688
MPA diameter, mm	8.91 ± 2.76	8.94 ± 2.72	0.967
Coronary fistula, *n* (%)	0 (0.0%)	1 (1.3%)	>0.999
Surgical Parameters			
First surgery type, *n* (%)			0.540
Pulmonary Balloon Valvuloplasty	0 (0.0%)	5 (6.6%)	
RVOT Reconstruction	11 (52.4%)	45 (59.2%)	
Open Pulmonary Valvotomy	8 (38.1%)	19 (25.0%)	
Off-Pump Hybrid Procedure	2 (9.5%)	7 (9.2%)	
B-T shunt, *n* (%)	6 (28.6%)	32 (42.1%)	0.261
CPB Time, min	54 ± 36	52 ± 44	0.894
Aortic Occlusion, *n* (%)	11 (52.4%)	45 (59.2%)	0.575

B-T shunt, Blalock-Taussig shunt; CPB, cardiopulmonary bypass; MPA, main pulmonary artery; MV, mitral valve; PA, pulmonary artery; RA, right atrium; RV, right ventricle; RVOT, right ventricular outflow tract; TV, tricuspid valve.

**Table 3:** Incidence of individual component events of the short-term composite.

Adverse Event	Total (*N* = 97)	Preterm (*n* = 21)	Term (*n* = 76)	*p*-value
Composite endpoint	27 (27.83%)	12 (57.14%)	15 (19.73%)	0.037
Adverse Events				0.880
Death	8 (29.63%)	3 (25.00%)	5 (33.33%)	
Malignant Arrhythmia	3 (11.11%)	2 (16.67%)	1 (6.67%)	
Postoperative Reintervention	5 (18.52%)	2 (16.67%)	3 (20.00%)	
Diaphragmatic Paralysis	1 (3.70%)	0 (0%)	1 (6.67%)	
MODS	4 (14.81%)	2 (16.67%)	2 (13.33%)	
Prolonged ICU Stay (>15 days)	3 (11.11%)	2 (16.67%)	1 (6.67%)	
Prolonged Hospital Stay (>30 days)	3 (11.11%)	1 (8.33%)	2 (13.33%)	

MODS, multiple organ dysfunction syndrome; ICU, intensive care unit.

Values are presented as *n* (%). *P*-values were calculated using Fisher's exact test.

We compared short-term and long-term outcomes between low birth weight (<2,500 g, *n* = 19) and normal birth weight (≥2,500 g, *n* = 78) infants. Notably, the short-term composite endpoint occurred significantly more often in the low birth weight group (63.2% vs. 19.2%, *p* < 0.001). For long-term outcomes, no significant differences were observed in final circulation type, reintervention rate, mortality, or the long-term adverse outcome composite (all *p* > 0.05) (Supplementary Table S2). Only 4 infants had a birth weight <2 kg. Two infants experienced adverse short-term events (one required postoperative reintervention, and another had a prolonged hospital stay). Two infants developed long-term adverse events (one required reintervention during follow-up, and one had a final circulation of 1.5-ventricle repair) (Supplementary Table S3).

### 3.2 Association between gestational age and short-term outcomes

**Figure 1:**
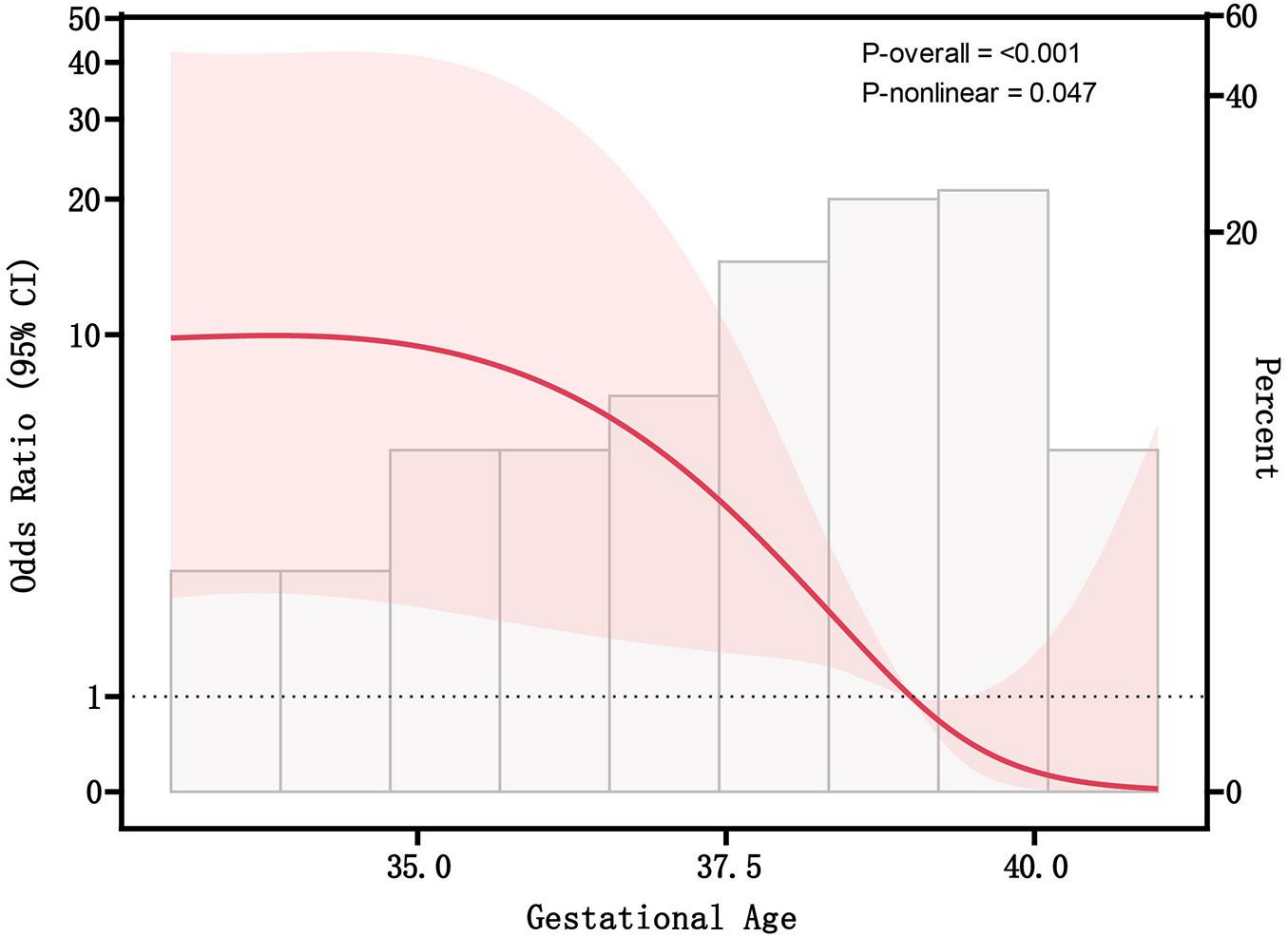
Predictive performance of models incorporating GA for short-term outcomes in neonates with PA/IVS. ROC curves illustrate the discriminative ability of Model 1 and Model 2 for predicting short-term outcomes.

Logistic regression analyses demonstrated a consistent and inverse association between GA and the risk of the composite short-term adverse outcome (Table 4). Each additional week of gestation was associated with significantly reduced odds of the outcome in all models. In the unadjusted model (Model 1), the odds decreased by 37% per week (OR 0.63, 95% CI 0.50–0.79; *p* < 0.001). This effect was strengthened following adjustment for covariates. The adjusted model (Model 2) showed a 49% reduction in odds per week (OR 0.51, 95% CI 0.41–0.75; *p* < 0.001). When GA was treated as a categorical variable, infants born at term exhibited markedly lower odds of the adverse outcome compared to preterm infants, with the most substantial effect observed in Model 2 (OR 0.14, 95% CI 0.04–0.46; *p* = 0.001). The discriminatory performance of the models improved progressively with increasing adjustment, as reflected in the area under the ROC curve (Figure 1). Collinearity diagnostics revealed no significant multicollinearity among the predictors (Supplementary Table S4), supporting the stability of the regression estimates.

**Table 4:** Logistic regression association between gestational age and short-term outcome.

**Characteristic**	**Model 1**			**Model 2**		
	**OR**	**95% CI**	***p*-value**	**OR**	**95% CI**	***p*-value**
Gestational age (continuous)	0.63	0.50, 0.79	<0.001	0.55	0.41, 0.75	<0.001
Gestational age						
<37	—	—		—	—	
≥37	0.18	0.07, 0.52	0.001	0.14	0.04, 0.46	0.001
P for trend			<0.001			<0.001

CI, Confidence Interval; OR, Odds Ratio.

Model 1: no covariates were adjusted.

Model 2: adjusted for Prenatal diagnosis, RV hypoplasia.

**Figure 2:**
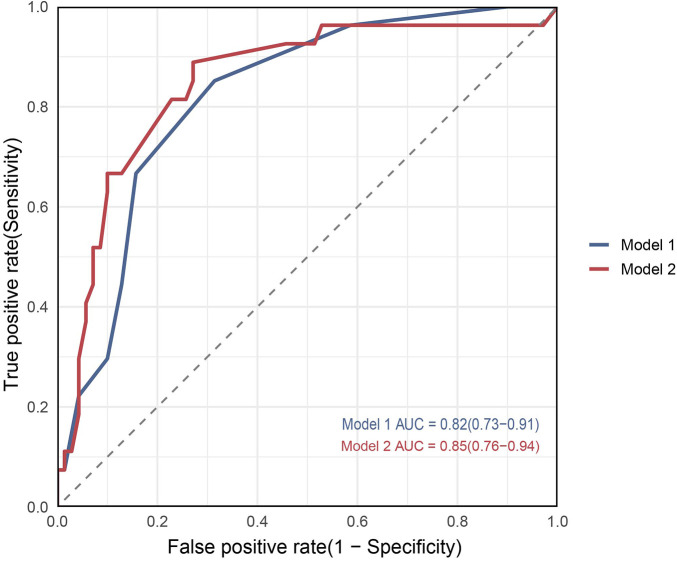
Dose-response relationship between GA and short-term poor outcome in neonates with PA/IVS. RCS plots show the unadjusted association (Model 1) of gestational age with the odds of short-term poor outcome.

A nonlinear association between GA and short-term adverse outcomes was identified using RCS regression (Figure 2). In the unadjusted model, the overall association was highly significant (*P*-overall < 0.001), with evidence of nonlinearity (*P*-nonlinea*r* = 0.047). To further characterize this nonlinearity, a two-piecewise logistic regression model was applied (Table 5), revealing an inflection point at 37.6 weeks (95% CI: 36.6–38.7). Below this threshold, GA was not significantly associated with short-term outcome risk (*p* = 0.919). Beyond this threshold infants had a 92% lower risk compared with those under 37.6 weeks (OR 0.08, 95% CI 0.02–0.42, *P* = 0.003). The threshold model provided a significantly better fit than the linear model (*p* = 0.002).

**Table 5:** Threshold effects of gestational Age and short-term poor prognosis.

**Model**	**OR (95% CI)**	***P*-value**
Model 1: Standard logistic regression	0.52 (0.35, 0.72)	<0.001
Model 2: Two-piecewise logistic regression		
Inflection point (weeks)	37.62 (36.57, 38.68)	
<37.62 weeks	1.03 (0.58, 1.83)	0.919
≥37.62 weeks	0.08 (0.02, 0.42)	0.003
Likelihood ratio test		0.002

CI, Confidence Interval; OR, Odds Ratio.

For long-term adverse outcomes, Cox regression analyses revealed no significant association with GA, whether modeled as a continuous or categorical variable, in any of the three adjusted models (Supplementary Table S6). Moreover, trend analysis showed no evidence of a dose-response relationship (*p* > 0.05).

In addition to GA, we also evaluated the independent role of birth weight on short-term and long-term outcomes. Birth weight was not independently associated with short-term outcomes after adjusting for GA and right ventricular hypoplasia (OR 0.33, *p* = 0.092; *P* for trend = 0.613), nor with long-term outcomes in any model (all *p* > 0.05) (Supplementary Table S5, S7).

### 3.3 Subgroup analyses

**Figure 3:**
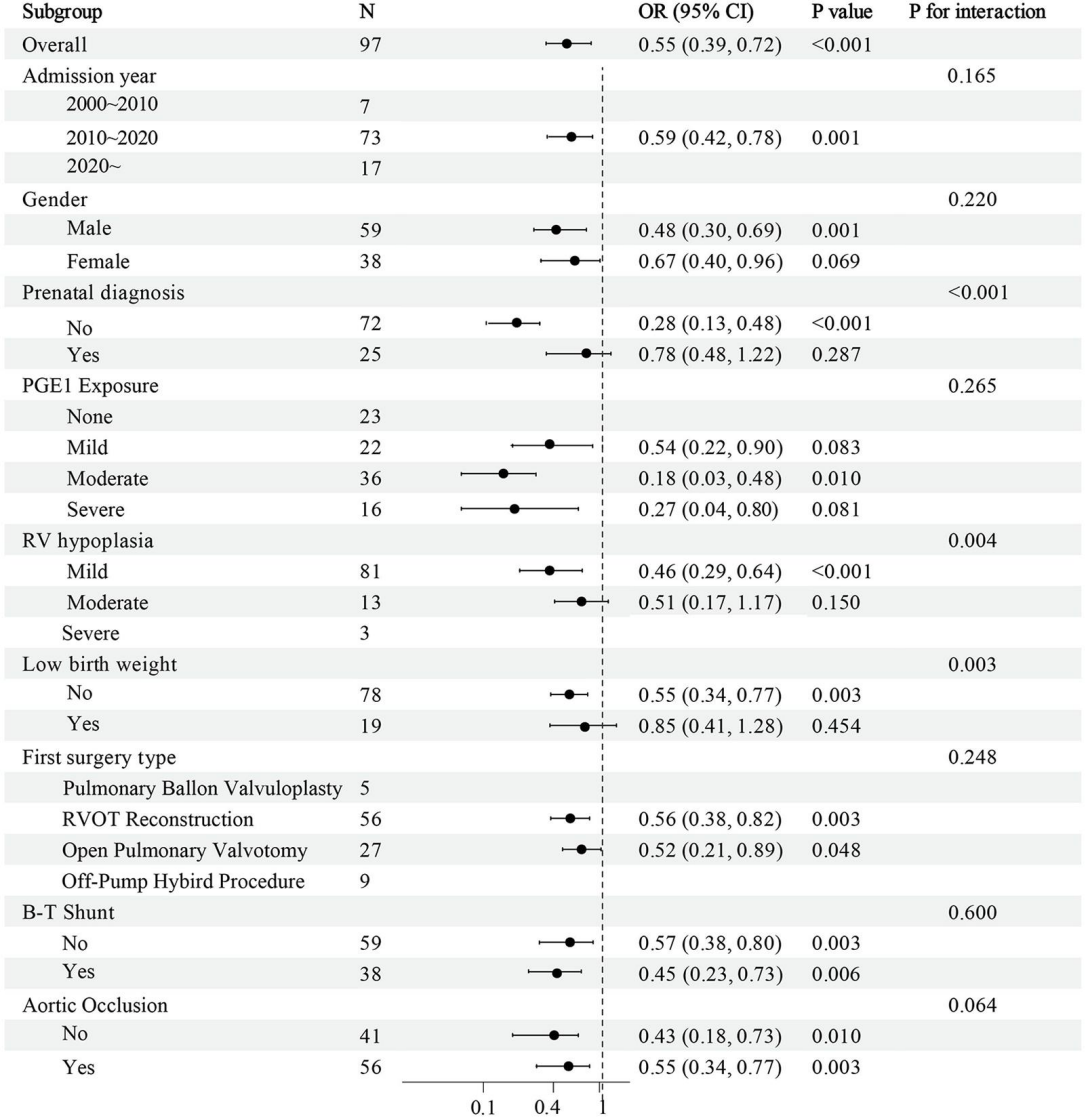
Subgroup analyses of the association between GA and short-term poor outcome in neonates with PA/IVS. Forest plot displays odds ratios (ORs) with 95% confidence intervals for the association across various patient subgroups. Significant effect modifications were observed for prenatal diagnosis, RV hypoplasia severity, and low birth weight status.

Subgroup analyses based on the fully adjusted model (Model 2) revealed that the protective effect of increasing GA against the short-term adverse outcome was consistent across most predefined subgroups, with significant effect modification observed for several factors (Figure 3). A strong interaction was identified with prenatal diagnosis status (*P* for interaction <0.001). The association was markedly stronger in infants without a prenatal diagnosis (OR: 0.28, 95% CI: 0.13–0.48; *p* < 0.001) compared to those with a prenatal diagnosis (OR: 0.78, 95% CI: 0.48–1.22; *p* = 0.287). Significant effect modification was also observed for the degree of RV hypoplasia (*P* for interaction = 0.004) and low birth weight status (*P* for interaction = 0.003). The inverse association was particularly pronounced in patients with mild RV hypoplasia (OR: 0.46, 95% CI: 0.29–0.64; *p* < 0.001) and in those with normal birth weight (OR: 0.32 95% CI: 0.15–0.54; *p* < 0.001). In contrast, no significant association was observed in infants with low birth weight (*p* = 0.454). No significant interaction was found for other subgroups (all *P* for interaction >0.05), indicating a consistent protective effect of GA within these strata.

## 4 Discussion

The principal finding of this study is the identification of a quantifiable, population-specific threshold at 37.6 weeks that dichotomizes short-term risk in PA/IVS infants undergoing biventricular repair. Below this threshold, GA demonstrated no significant association with outcomes (*p* = 0.919), while beyond it, each additional week conferred a 92% reduction in adverse event odds (OR 0.08, *p* = 0.003). This nonlinear relationship, with the threshold model significantly outperforming linear approaches (*p* = 0.002), indicates that the physiological benefits of fetal maturation in this specific population are not accrued linearly but rather become manifest after a critical maturity point. Rather than simply validating the conventional “early term” concept, our data precisely quantify the onset of risk reduction, providing a data-driven benchmark for clinical decision-making in this selected cohort.

The threshold at 37.6 weeks corresponds with key stages of fetal cardiovascular maturation. Preterm birth affects PA/IVS outcomes through several mechanisms. First, preterm birth leads to aberrant pulmonary vascular development, manifesting as pulmonary hypertension and increased vascular resistance and stiffness, thereby elevating right ventricular afterload (13, 14). Most critically, surfactant deficiency causes alveolar collapse and ventilation-perfusion mismatch, which severely compromises postoperative pulmonary oxygenation, increases the risk of serious complications and is associated with lower survival rates following surgery (15–17). This mechanism likely underlies why GA emerged as the strongest predictor in our study. Before 37 weeks of gestation, the cardiovascular system remains functionally and morphologically immature, with elevated pulmonary vascular resistance, reduced myocardial contractile reserve, and impaired adaptive capacity to hemodynamic stress (13, 18). Cheung PY et al. suggest preterm neonates with complex CHD and early open-heart surgery had significant mortality and morbidity at 2-years (19). The RV dependent circulation places additional demands on an already immature myocardium, explaining the dramatic threshold effect at 37.6 weeks (20). Our findings both confirm and extend previous observations. Costello et al. reported increased mortality with early term delivery (37–38 weeks) in a large heterogeneous cohort of congenital heart disease patients6, while our threshold analysis provides greater precision. The discrepancy with Savorgnan et al.'s null findings at 37–38 weeks likely stems from their inclusion of diverse cardiac lesions with varying cyanosis and hemodynamic impact (21). In summary, GA demonstrates a distinct threshold effect on short-term prognosis, with the inflection at 37.6 weeks offering a potential benchmark for optimizing perinatal management and surgical timing.

Preterm infants in our cohort had a higher rate of prenatal diagnosis. A likely explanation is that more severe anatomical abnormalities are more readily detected, prompting closer obstetric monitoring and, if fetal distress appears, earlier delivery (22). Thus, preterm birth may reflect greater disease severity or obstetric intervention rather than being an isolated event. However, our data argue against disease severity as the sole explanation: GA remained independently associated with outcomes after adjusting for RV hypoplasia (Model 2), and the significant interaction between GA and RV hypoplasia (*P* = 0.004) showed that the protective effect of later gestation was most pronounced in milder anatomy, while severe hypoplasia limited the benefit. The clear threshold at 37.6 weeks, coinciding with cardiovascular maturation milestones.

Subgroup analyses revealed that the protective effect of later gestation was more pronounced in cases without prenatal diagnosis and those with milder RV hypoplasia, suggesting that prenatal planning may partially compensate for prematurity, while preserved compensatory mechanisms in milder anatomy benefit more from gestational maturity (23–25). However, the generalizability of this finding is limited, as prenatal diagnosis rates depend on multiple factors—including screening program efficiency, operator expertise and inter-center variability. The differential effect based on birth weight status underscores the importance of overall fetal development. Intrauterine growth restriction may limit the capacity to benefit from gestational maturity and has been independently associated with increased perioperative mortality (26, 27). However, in our analysis, after correcting for GA and right ventricular hypoplasia, the association between birth weight and short-term outcomes weakened and lost statistical significance, indicating that the apparent benefit of higher birth weight is primarily driven by gestational maturity rather than birth weight.

In contrast, GA showed no association with long-term outcomes. This dissociation suggests that early postoperative adaptation differs from long-term prognosis, which is likely governed by sustained anatomical and hemodynamic factors (4, 11). Competing risks and survivor bias may also contribute: infants who survived to long-term follow-up represent a selected cohort, potentially attenuating the measurable impact of GA, and the limited number of long-term events reduced statistical power.

### 4.1 Clinical implications

For PA/IVS pregnancies with anticipated biventricular repair, delivery timing should incorporate the 37.6-week threshold when obstetric conditions permit. Later delivery within the term window confers incremental benefit, balancing the gains in fetal maturity against established obstetric risks of late-term pregnancies (28). The predictive model incorporating GA offers a tool for preoperative risk stratification in BiV repair, and high-risk patients may benefit from enhanced perioperative monitoring or modified surgical approaches. The threshold effect also informs family counseling.

### 4.2 Limitations

Several limitations of this study should be considered. First, as the inclusion criteria were restricted to PA/IVS patients who underwent biventricular repair, our findings may not be generalizable to those managed with univentricular palliation or other palliative procedures. Second, the exclusion of patients with major extracardiac anomalies or chromosomal abnormalities limits the applicability of our results to these complex subgroups. Furthermore, although this retrospective single-center study adjusted for known confounders in multivariable models, the potential for residual confounding and selection bias cannot be fully eliminated. Additionally, as the study population consisted primarily of Chinese patients, caution is warranted when extrapolating the findings to other ethnic groups and health care systems due to potential differences in genetic background and clinical management. Although the sample size is relatively large for this rare condition, certain subgroup analyses may still have been underpowered, potentially leading to null findings. Future prospective, multi-center studies involving larger and more diverse cohorts are needed to validate and generalize these results.

## 5 Conclusion

This study identifies 37.6 weeks as a critical GA threshold for short-term outcomes in PA/IVS patients undergoing BiV repair, beyond which each additional week was associated with progressively lower risks. This protective effect was especially evident among infants without a prenatal diagnosis, those with mild right ventricular hypoplasia, and those with normal birth weight. In contrast, no significant association was found between GA and long-term outcomes. Clinically, these findings suggest that delivery after 37.6 weeks may optimize short-term recovery without compromising long-term prognosis.

## Data Availability

The original contributions presented in the study are included in the article/Supplementary Material, further inquiries can be directed to the corresponding author/s.

## References

[1] Cheung EW Mastropietro CW Flores S Amula V Radman M Kwiatkowski D. Procedural outcomes of pulmonary atresia with intact ventricular septum in neonates: a multicenter study. Ann Thorac Surg. (2023) 115(6):1470–7. 10.1016/j.athoracsur.2022.07.055 36070807

[2] Jaggers J Winlaw D Fuller S Sethi N Kochilas L Adachi I. 2025 American association for thoracic surgery congenital cardiac surgery working group-expert consensus document on the management of patients with pulmonary atresia with intact ventricular septum. J Thorac Cardiovasc Surg. (2025) 170(2):336–52. 10.1016/j.jtcvs.2025.03.034 40320005

[3] Sukhavasi A McHugh-Grant S Glatz AC Mondal A Griffis H Burnham N. Pulmonary atresia with intact ventricular septum: intended strategies. J Thorac Cardiovasc Surg. (2022) 164(5):1277–88. 10.1016/j.jtcvs.2021.11.104 35414413

[4] Sugitani Y Muneuchi J Watanabe M Matsuoka R Doi H Ezaki H. Late adverse events in patients with pulmonary atresia with intact ventricular septum after valvuloplasty. Ann Thorac Surg. (2022) 113(6):2072–8. 10.1016/j.athoracsur.2021.04.003 33864755

[5] Zou M Dong S Liu S Du C Sun Y Dong J. Influencing factors of prognosis in children with pulmonary atresia with intact ventricle septum after transthoracic balloon dilation of pulmonary valve and construction of a nomograph prediction model. Biotechnol Genet Eng Rev. (2023) 40(4):4328–40. 10.1080/02648725.2023.2210448 37154016

[6] Costello JM Pasquali SK Jacobs JP He X Hill KD Cooper DS. Gestational age at birth and outcomes after neonatal cardiac surgery: an analysis of the society of thoracic surgeons congenital heart surgery database. Circulation. (2014) 129(24):2511–7. 10.1161/CIRCULATIONAHA.113.005864 24795388 PMC4276251

[7] Savorgnan F Elhoff JJ Guffey D Axelrod D Buckley JR Gaies M. Relationship between gestational age and outcomes after congenital heart surgery. Ann Thorac Surg. (2021) 112(5):1509–16. 10.1016/j.athoracsur.2020.08.027 33080235 PMC8052379

[8] Steurer MA Schuhmacher K Savla JJ Banerjee M Chanani NK Eckhauser A. Fetal growth and gestational age improve outcome predictions in neonatal heart surgery. J Thorac Cardiovasc Surg. (2022) 164(6):2003–2012.e1. 10.1016/j.jtcvs.2022.05.022 35750509

[9] Best KE Tennant PWG Rankin J. Survival, by birth weight and gestational age, in individuals with congenital heart disease: a population-based study. J Am Heart Assoc. (201721) 6(7):e005213. 10.1161/JAHA.116.005213 28733436 PMC5586271

[10] Puchalski MD Lui GK Miller-Hance WC Brook MM Young LT Bhat A. Guidelines for performing a comprehensive transesophageal echocardiographic: examination in children and all patients with congenital heart disease: recommendations from the American society of echocardiography. J Am Soc Echocardiogr. (2019) 32(2):173–215. 10.1016/j.echo.2018.08.016 30579694

[11] Deshaies C Trottier H Khairy P Al-Aklabi M Beauchesne L Bernier P-L; Canadian Congenital Cardiac Collaborative (4C) Tricuspid intervention following pulmonary valve replacement in adults with congenital heart disease. J Am Coll Cardiol. (2020) 75(9):1033–43. 10.1016/j.jacc.2019.12.053 32138963

[12] Wright LK Knight JH Thomas AS Oster ME St Louis JD Kochilas LK. Long-term outcomes after intervention for pulmonary atresia with intact ventricular septum. Heart. (2019) 105(13):1007–13. 10.1136/heartjnl-2018-314124 30712000 PMC6571047

[13] Lewandowski AJ Levy PT Bates ML McNamara PJ Nuyt AM Goss KN. Impact of the vulnerable preterm heart and circulation on adult cardiovascular disease risk. Hypertension. (2020) 76(4):1028–37. 10.1161/HYPERTENSIONAHA.120.15574 32816574 PMC7480939

[14] Mulchrone A Bellofiore A Douwes JM Duong N Beshish AG Barton GP. Impaired right ventricular-vascular coupling in young adults born preterm. Am J Respir Crit Care Med. (2020) 201(5):615–8. 10.1164/rccm.201904-0767LE 31697579 PMC7047464

[15] Whitsett JA Weaver TE. Hydrophobic surfactant proteins in lung function and disease. N Engl J Med. (2002) 347(26):2141–8. 10.1056/NEJMra022387 12501227

[16] Yammine S Schmidt A Sutter O Fouzas S Singer F Frey U. Functional evidence for continued alveolarisation in former preterms at school age? Eur Respir J. (2016) 47(1):147–55. 10.1183/13993003.00478-2015 26493788

[17] Gaynor JW Parry S Moldenhauer JS Simmons RA Rychik J Ittenbach RF. The impact of the maternal-foetal environment on outcomes of surgery for congenital heart disease in neonates. Eur J Cardiothorac Surg. (2018) 54(2):348–53. 10.1093/ejcts/ezy015 29447332 PMC6454438

[18] Goss KN Beshish AG Barton GP Haraldsdottir K Levin TS Tetri LH. Early pulmonary vascular disease in young adults born preterm. Am J Respir Crit Care Med. (2018) 198(12):1549–58. 10.1164/rccm.201710-2016OC 29944842 PMC6298636

[19] Cheung P-Y Hajihosseini M Dinu IA Switzer H Joffe AR Bond GY. Outcomes of preterm infants with congenital heart defects after early surgery: defining risk factors at different time points during hospitalization. Front Pediatr. (2021) 8:616659. 10.3389/fped.2020.616659 33585367 PMC7876369

[20] Telles F McNamara N Nanayakkara S Doyle MP Williams M Yaeger L. Changes in the preterm heart from birth to young adulthood: a meta-analysis. Pediatrics. (2020) 146(2):e20200146. 10.1542/peds.2020-0146 32636236

[21] Goldshtrom N Vasquez AM Chaves DV Bateman DA Kalfa D Levasseur S. Outcomes after neonatal cardiac surgery: the impact of a dedicated neonatal cardiac program. J Thorac Cardiovasc Surg. (2023) 165(6):2204–2211.e4. 10.1016/j.jtcvs.2022.06.013 35927084

[22] Donofrio MT Moon-Grady AJ Hornberger LK Copel JA Sklansky MS Abuhamad A. Diagnosis and treatment of fetal cardiac disease: a scientific statement from the American Heart Association. Circulation. (2014) 129(21):2183–242. 10.1161/01.cir.0000437597.44550.5d 24763516

[23] Zhang Y Wang J Zhao J Huang G Liu K Pan W. Current status and challenges in prenatal and neonatal screening, diagnosis, and management of congenital heart disease in China. Lancet Child Adolesc Health. (2023) 7(7):479–89. 10.1016/S2352-4642(23)00051-2 37301215

[24] Rako ZA Kremer N Yogeswaran A Richter MJ Tello K. Adaptive versus maladaptive right ventricular remodelling. ESC Heart Fail. (2023) 10(2):762–75. 10.1002/ehf2.14233 36419369 PMC10053363

[25] Liava'a M Brooks P Konstantinov I Brizard C d'Udekem Y. Changing trends in the management of pulmonary atresia with intact ventricular septum: the Melbourne experience. Eur J Cardiothorac Surg. (2011) 40(6):1406–11. 10.1016/j.ejcts.2011.02.036 21561788

[26] Gunasekara CM Moynihan K Sudhakar A Sunil GS Kotayil BP Bayya PR. Neonatal cardiac surgery in low resource settings: implications of birth weight. Arch Dis Child. (2020) 105(12):1140–5. 10.1136/archdischild-2020-319161 32718929

[27] Henmi S Essa Y Öztürk M Tongut A Desai M Yerebakan C. Cardiovascular surgery in very low birth weight (≤1500g) neonates. Eur J Cardiothorac Surg. (2022) 63(1):ezac552. 10.1093/ejcts/ezac552 36469322

[28] Källén K Norman M Elvander C Bergh C Sengpiel V Hagberg H. Maternal and perinatal outcomes after implementation of a more active management in late- and postterm pregnancies in Sweden: a population-based cohort study. PLoS Med. (2025) 22(1):e1004504. 10.1371/journal.pmed.1004504 39820829 PMC11737695

