# Supplementary material for: Gestational age as predictor of postoperative prognosis in neonates with pulmonary atresia with intact ventricular septum undergoing biventricular repair
Source: Front Cardiovasc Med. 2026 Jun 22;13:1813144. doi: 10.3389/fcvm.2026.1813144 (PMC13333623; doi:10.3389/fcvm.2026.1813144)
Supplement: Supplementary file 1 [file Datasheet1.docx]

**Supplement Materials:**

**Classification of Variables**

(1) Atrial septal defect (ASD) : categorized based on echocardiographic measurements as small (<3 mm), moderate (3–6 mm), or large (>6 mm);

(2) Patent ductus arteriosus (PDA) : classified according to ductal diameter and shunt direction as trivial (<2 mm), moderate (2–4 mm), or large (>4 mm);

(3) Subpulmonic stenosis: including sub-valvular fibrous ring and muscular bundle obstruction;

(4) Coronary artery anomalies: comprising right ventricle-dependent coronary circulation (RVDCC) and coronary artery fistula;

(5) Complex cardiac anomalies specified in the exclusion criteria refer to malformations requiring complex surgical reconstruction, such as transposition of the great arteries, complete atrioventricular septal defect, and Ebstein's anomaly.

(6) PGE1 Exposure was categorized by cumulative dosage into four grades: grade 0 (none), grade 1 (low-intensity: ≤0.05 μg/kg/min for ≤7 days), grade 2 (moderate-intensity: exposure between grade 1 and 3), and grade 3 (high-intensity: >0.05 μg/kg/min for >7 days), so as to simplify assessment and reduce classificatory complexity

**The measurement of Echocardiographic**

(1) Right ventricular dimensions (in mm), measured at end-diastole in the apical four-chamber view as the maximal transverse diameter at the level of the tricuspid annulus;

(2) Tricuspid valve annulus *z*-score, calculated as (measured value - mean normal value) / standard deviation, with reference norms based on Pediatric Diagnostic Ultrasound (2nd edition)^1^;

(3) Degree of right ventricular hypoplasia, graded as mild, moderate, or severe according to the modified Bull’s classification, based on the involvement of the inflow, trabecular and outlet portions, as well as basal segment diameter relative to normative standards^2^.

**Operation strategies and techniques**

The modified Blalock-Taussig shunt was performed under systemic heparinization. The right subclavian artery was clamped and an arteriotomy made, followed by an end-to-side anastomosis using a graft with continuous suture. Similarly, the right pulmonary artery was clamped and opened, and the graft anastomosed in an end-to-side fashion. Upon release of the clamps, the anastomotic sites were inspected and confirmed to be secure.

Transcatheter pulmonary valve balloon dilation was initiated via femoral venous access. After sheath placement, a catheter was advanced to the center of the atretic pulmonary valve. A radiofrequency perforation wire was used to penetrate the valve under fluoroscopic guidance. Once the wire entered the pulmonary artery, it was exchanged for a guidewire to establish stable access. An appropriately sized balloon was then advanced and inflated across the valve until the waist sign resolved. Post-dilation angiography confirmed adequate valve opening without complication before sheath removal and haemostasis.

For right ventricular outflow tract (RVOT) reconstruction, a median sternotomy was performed and cardiopulmonary bypass established. After aortic cross-clamping, the main pulmonary artery was incised longitudinally and the pulmonary valve leaflets were divided. A vertical right ventriculotomy was made, and obstructive muscle bundles were excised. The RVOT and pulmonary artery were augmented with a pericardial patch using continuous suture. Tricuspid valve competence was assessed by saline testing prior to weaning from bypass.

Open pulmonary valvotomy was carried out via median sternotomy. Based on individual hemodynamics, cardiopulmonary bypass was omitted in selected cases. The distal pulmonary artery was controlled, and a longitudinal arteriotomy performed. The adherent valve leaflets were incised and dilated to approximately 3.5 mm. The arteriotomy was then closed with a continuous suture technique.

The hybrid off-pump procedure was performed through a median sternotomy. Purse-string sutures were placed on the right ventricular free wall. Under transesophageal echocardiography guidance, percutaneous access was achieved, the atretic valve perforated, and a balloon catheter advanced and inflated to establish antegrade flow. The access site was secured with previously placed sutures and the wound closed in layers.
**Supplement References:**

1. Lewandowski AJ, Levy PT, Bates ML, McNamara PJ, Nuyt AM, Goss KN. Impact of the vulnerable preterm heart and circulation on adult cardiovascular disease risk. Hypertension. (2020) 76(4):1028–37. doi: 10.1161/HYPERTENSIONAHA.120.15574
2. Bull C, De Leval MR, Mercanti C, Macartney FJ, Anderson RH. Pulmonary atresia and intact ventricular septum: a revised classification. Circulation. (1982) 66(2):266–72. doi: 10.1161/01.cir.66.2.266

**Supplement Table:**

**Table S1. Patient supplement demographics and baseline characteristics**

| **Characteristic** | **Gestational Age Group** | | | ***p*-value** | |
| --- | --- | --- | --- | --- | --- |
|  | **˂ 37 weeks**  **N = 21** | **≥ 37 weeks**  **N = 76** | |  |  |
| ***Perioperative Characteristic*** |  |  | |  | |
| First treatment weight, kg | 3.34 ± 1.15 | 3.95 ± 1.69 | | 0.059 | |
| Oxygen saturation, % | 74 ± 13 | 73 ± 15 | | 0.843 | |
| PGE1 Exposure, n (%) | | | | >0.999 | |
| None | 5 (23.8%) | 18 (23.7%) | |  | |
| Mild | 5 (23.8%) | 17 (22.4%) | |  | |
| Moderate | 8 (38.1%) | 28 (36.8%) | |  | |
| Severe | 3 (14.3%) | 13 (17.1%) | |  | |
| Ross class, n (%) |  |  | | 0.859 | |
| Ⅰ | 0 (0.0%) | 1 (1.3%) | |  | |
| Ⅱ | 0 (0.0%) | 2 (2.6%) | |  | |
| Ⅲ | 18 (85.7%) | 65 (85.5%) | |  | |
| Ⅳ | 3 (14.3%) | 8 (10.5%) | |  | |
| ASD diameter, n (%) |  |  | | 0.245 | |
| Large | 4(19.0%) | 29(38.2%) | |  | |
| Moderate | 16(76.2%) | 43(56.6%) | |  | |
| Small | 1(4.8%) | 4(5.3%) | |  | |
| PDA diameter, n (%) |  |  | | 0.581 | |
| Moderate | 12(57.1%) | 52(68.4%) | |  | |
| Large | 4(19.0%) | 9(11.8%) | |  | |
| Trivial | 5(23.8%) | 15(19.7%) | |  | |
| ASD shunt direction, n (%) |  |  | | 0.460 | |
| Bidirectional | 7(33.3%) | 19(25.0%) | |  | |
| Right to Left | 0(0.0%) | 4(5.3%) | |  | |
| Left to Right | 14(66.7%) | 53(69.7%) | |  | |
| PDA shunt direction, n (%) |  |  | | 0.143 | |
| Bidirectional | 1(4.8%) | 1(1.3%) | |  | |
| Right to Left | 0(0.0%) | 10(13.2%) | |  | |
| Left to Right | 20(95.2%) | 65(85.5%) | |  | |
| ***Laboratory Parameters*** | | | | | |
| Hb, g/L | 142 ± 31 | 156 ± 27 | | 0.064 | |
| Plt, ×10⁹/L | 274 ± 101 | 256 ± 100 | | 0.481 | |
| Pt, s | 17.05 ± 4.21 | 15.75 ± 2.57 | | 0.188 | |
| ALT, U/L | 17 ± 15 | 21 ± 21 | | 0.349 | |
| AST, U/L | 68 ± 64 | 52 ± 53 | | 0.312 | |
| Cr, μmol/L | 48 ± 18 | 57 ± 26 | | 0.080 | |
| CK, U/L | 415 ± 591 | 467 ± 477 | | 0.714 | |
| CK-MB, U/L | 64 ± 94 | 42 ± 50 | | 0.310 | |
| LDH, U/L | 466 ± 270 | 483 ± 282 | | 0.795 | |
| HBDH, U/L | 354 ± 189 | 346 ± 188 | | 0.860 | |
| ***Long-term Outcomes*** | | | | | |
| Follow-up Time, year | 5.75±4.27 | | 6.95±4.80 | | 0.809 |
| Mortality, n (%) | 2 (9.5%) | 6 (7.9%) | | >0.999 | |
| Cardiac death, n (%) | 2 (100.0%) | 5 (83.3%) | | >0.999 | |
| Final circulation type, n (%) |  |  | | 0.150 | |
| BiV circulation | 17 (81.0%) | 67(88.2%) | |  | |
| 1.5V circulation | 3 (14.3%) | 9(11.8%) | |  | |
| SV circulation | 1 (4.8%) | 0 (0.0%) | |  | |
| Reintervention, n (%) | 6 (28.6%) | 27 (25.5%) | | 0.552 | |
| Long-term Adverse Outcome, n (%) | 10 (47.6%) | 36 (47.4%) | | 0.984 | |

**Abbreviations:** BiV, biventricular; 1.5V. one and a half ventricular; SV, single ventricle; PT, prothrombin time; PGE1, prostaglandin E1; Plt, platelet count; PT, prothrombin time; Cr, creatinine; CK, creatine kinase; CK-MB, creatine kinase-MB; Hb, hemoglobin; HBDH, α-hydroxybutyrate dehydrogenase; LDH, lactate dehydrogenase; ALT, alanine aminotransferase; AST, aspartate aminotransferase.

**Table S2. Incidence of Individual Component Events of the Short-term and Long-term Composite between low birth weight (<2500 g) and birth weight ≥2500 g**

| Adverse Event | Low Weight (n=19) | Normal Weight (n=78) | p-value |
| --- | --- | --- | --- |
| ***Short-term Outcomes*** |  |  |  |
| Short-term Composite endpoint | 12（63.2%） | 15（19.20%） | <0.001 |
| Short-term Adverse Events |  |  | <0.001 |
| Death | 1(5.26%) | 7(8.97%) |  |
| Malignant Arrhythmia | 1(5.26%) | 2(2.56%) |  |
| Postoperative Reintervention | 2(10.53%) | 3(3.85%) |  |
| Diaphragmatic Paralysis | 0(0.00%) | 1(1.28%) |  |
| MODS | 3(15.79%) | 1(1.28%) |  |
| Prolonged ICU Stay (>15 days) | 3(15.79%) | 0(0.00%) |  |
| Prolonged Hospital Stay (>30 days) | 2(10.53%) | 1(1.28%) |  |
| ***Long-term Outcomes*** |  |  |  |
| Follow-up Time, year | 6.94±5.18 | 6.63±4.60 | 0.814 |
| Final circulation type, n (%) |  |  | 0.786 |
| BiV circulation | 16(84.21%) | 68(87.18%) |  |
| 1.5V circulation | 3(15.80%) | 9(11.54%) |  |
| SV circulation | 0(0.00%) | 1(1.28%) |  |
| Reintervention, n (%) | 8(42.11%) | 25(32.05%) | 0.407 |
| Mortality, n (%) | 2(10.53%) | 6(7.69%) | 0.687 |
| Long-term Adverse Outcome, n (%) | 11(57.89%) | 35(44.87%) | 0.308 |

**Table S3. Incidence of Individual Component Events of the Short-term and Long-term Composite in birth weight <2000 g infants**

| **Patients** | **Birth Weight** | **Short-term Outcome** | **Long-term Outcome** |
| --- | --- | --- | --- |
| **Patient 1** | 1.58kg | Prolonged Hospital Stay | —— |
| **Patient 2** | 1.10kg | Postoperative Reintervention | —— |
| **Patient 3** | 1.90kg | —— | Final 1.5V repair |
| **Patient 4** | 1.85kg | —— | Reintervention |

**Table S4. The variance inflation factors of the variables included in the model**

| Variable | VIF |
| --- | --- |
| Gestational age | 1.150 |
| RV hypoplasia | 1.022 |
| Prenatal diagnosis | 1.129 |

**Table S5. Logistic regression Association between birth weight and Short-term outcome.**

| **Characteristic** | **Model 3** | | | **Model 4** | | |
| --- | --- | --- | --- | --- | --- | --- |
|  | **OR** | **95% CI** | **p-value** | **OR** | **95% CI** | **p-value** |
| **Birth Weight (continuous)** | 1.05 | 0.90, 1.23 | 0.512 | 1.32 | 0.66, 2.63 | 0.430 |
| **Birth Weight** |  |  |  |  |  |  |
| < 2500g | — | — |  | — | — |  |
| ≥ 2500g | 0.14 | 0.05, 0.41 | <0.001 | 0.33 | 0.09, 1.20 | 0.092 |
| **P for trend** |  |  | 0.024 |  |  | 0.613 |

**Abbreviations:** CI = Confidence Interval, OR = Odds Ratio

Model 3: no covariates were adjusted

Model 4: adjusted for Gestational age, RV hypoplasia

**Table S6. Cox regression Association between gestational age and long-term outcome.**

| **Characteristic** | **Model 1** | | | **Model 2** | | |
| --- | --- | --- | --- | --- | --- | --- |
|  | **HR** | **95% CI** | **p-value** | **HR** | **95% CI** | **p-value** |
| Gestational age (continuous) | 0.94 | 0.85, 1.04 | 0.244 | 0.93 | 0.82, 1.04 | 0.189 |
| Gestational age |  |  |  |  |  |  |
| Q1 | — | — |  | — | — |  |
| Q2 | 0.91 | 0.38, 2.16 | 0.839 | 0.85 | 0.35, 2.10 | 0.729 |
| Q3 | 0.83 | 0.39, 1.78 | 0.641 | 0.83 | 0.36, 1.89 | 0.652 |
| Q4 | 0.42 | 0.11, 1.58 | 0.197 | 0.38 | 0.09, 1.55 | 0.178 |
| P for trend |  |  | 0.617 |  |  | 0.595 |

**Abbreviations:** CI = Confidence Interval, HR = Hazard Ratio

The Models established same to Table 4.

**Table S7. Cox regression Association between birth weight and long-term outcome.**

| **Characteristic** | **Model 1** | | | **Model 2** | | |
| --- | --- | --- | --- | --- | --- | --- |
|  | **HR** | **95% CI** | **p-value** | **HR** | **95% CI** | **p-value** |
| Birth Weight (continuous) | 1.00 | 0.93,1.09 | 0.85 | 1.28 | 0.81,2.03 | 0.287 |
| Birth Weight |  |  |  |  |  |  |
| Q1 | — | — |  | — | — |  |
| Q2 | 0.90 | 0.36, 2.22 | 0.814 | 0.35 | 0.09, 1.40 | 0.136 |
| Q3 | 1.04 | 0.48, 2.27 | 0.917 | 0.90 | 0.41, 1.99 | 0.794 |
| Q4 | 0.64 | 0.20, 2.04 | 0.447 | 0.61 | 0.19, 1.95 | 0.406 |
| P for trend |  |  | 0.545 |  |  | 0.391 |

**Abbreviations:** CI = Confidence Interval, HR = Hazard Ratio

The Models established same to Table S4.
